# Supplementary material for: Phylogeny, time divergence, and historical biogeography of the South American Liolaemus alticolor-bibronii group (Iguania: Liolaemidae)
Source: PeerJ. 2018 Feb 20;6:e4404. doi: 10.7717/peerj.4404 (PMC5824678; doi:10.7717/peerj.4404)
Supplement: File S1 [file peerj-06-4404-s001.pdf]

Supplemental File S1

List of specimens studied and accession number of GeneBank from the sequences downloaded.

Museum acronyms correspond to Scientific Collections described as follow:

AMNH: American Museum of Natural History; **BYU:** Bean Life Science Museum at Brigham Young University**; CBF:** Colección Boliviana de Fauna; **CBGR:** Colección Herpetológica del Centro de Biodiversidad y Genética de Cochabamba; **CMNH:** Carnegie Museum of Natural History**; CORBIDI:** Centro de Ornitologia y Biodiversidad**; FML:** Fundación Miguel Lillo; **FMNH:** Field Museum of Natural History; **IBIGEO-R:** Reptiles section of the Herpetological Collection of the Instituto de BIo y Geociencias del NOA. **LJAMM-CNP:** Centro Nacional Patagónico de Puerto Madryn; **MACN:** Museo Argentino de Ciencias Naturales; **MCN**: Museo de Ciencias Naturales, Universidad Nacional de Salta; **MIC**: Miguel I. Christie, private collection; **MLP.S**: Museo de la Plata; **MNHN:** Museo Nacional de Historia Natural de Santiago; **MUSM:** Museo de Historia Natural de San Marcos; **MVZ**: Museum of Vertebrate Zoology, University of California; **MZUC:** Museo de Zoologia, Universidad de Concepción**; SDSU**: San Diego State University; **UNMDP:** Colección Herpetológica de la Universidad de Mar del Plata; **UNSJ:** Colección Herpetológica de la Facultad de Ciencias Exactas y Naturales de la Universidad Nacional de San Juan; **USNM:** Smithsonian Institution, National Museum of Natural History. BB correspond to field numbers, these specimens will be incorporated to MACN. PMC correspond to field numbers, these specimens will be incorporated to IBIGEO-R.

List of specimens studied for morphological characters. Numbers between parentheses indicate number of specimens under the same batch.

Liolaemus alticolor-**BOLIVIA: Departamento de La Paz**: Tiaguanacu, MCZR 169064 (lectotype), MCZ–R 7287 (paralectotype); Rio Huarocondo, MCZ 12409; Tiahuanaco, near Lake Titicaca, MCZR 128518–525; Tiahuanaco, CBF 2925, 2893–2896.

Liolaemus abdalai-**ARGENTINA: Provincia de Neuquén**: Ruta Provincial 23.8 km N de Pilolil, Orillas del Rio Aluminé, 39°22’29”S, 70°57’21”W, MCN 2741 (holotype), MCN 2739–40, 2742–43, FML 7843–44; Ruta Provincial 11, 0.2 km al oeste de Arroyo Remecó, 39°02’S; 71°21’W, MCN 2744–50; Arroyo Quilanlahue, Parque Nacional Lanin, Lacar, FML 1776.

Liolaemus aparicioi- **BOLIVIA. Departamento de La Paz.** Jupapina CBF 2917, 2998-99, 3017, 3180-81.

Liolaemus araucaniensis- **CHILE. Region de La Araucania.** FML 2702(29). FMNH 208980; 206974; 206985; 206987; 206988.995: 206998-7000; 208963.

Liolaemus bibronii- **ARGENTINA: Provincia de Santa Cruz**: Gruta de Lourdes, 2 km E Ruta Provincial 281, 11.2 km NW Puerto Deseado, FML 10106-107.

Liolaemus bitaeniatus- **ARGENTINA: Provincia de Catamarca**: Agua de las Palomas, FML 1932, 3593; Quebrada Peña La Horqueta - Distrito Espinillo, FML 6347; Confluencia de ríos Quebrada y Candado, Andalgalá, FML 7137– 38. **Provincia de Salta**: San Fernando de Escoipe, Chicoana FML 1655 (6). **Provincia de Tucumán**: Sierra de Medina, FML 2237 (2), 2345 (2); Dique La Angostura, El Mollar, FML 2384 (4), 2499 (2); El Mollar, FML 2462, 2475 (5); Cerro Las Botijas, Sierra de Medina, MCN 900– 901; SDSU 3569.

Liolaemus chaltin- **ARGENTINA: Provincia de Jujuy**: Departamento Cochinoca: 3 km NO de Abrapampa, FML 1461 (5); Abrapampa FML 1871 (4), 2513 (2); Ruta Provincial 71, 4.2 km W de Abrapampa, 22°42’24.4”S, 65°43’12.4”W, FML 9874 (holotype); 2 km N Abrapampa, 23°19’673”S, 66°05’399”W, MCN 235; 2.9 km de la intersección entre ruta 7 y 71, camino a Cochinoca, 6.8 km W de Abra Pampa, 22°45’59.4”S, 65°44’54.7”W, MCN 2221–31; SDSU 3572.

Liolaemus chungara- **CHILE: Región de Arica y Parinacota**: Entrada a Putre, 18°10’53.4”S, 69°31’58.6”W, 3874 m, FML 26505 (holotype), FML 26506 (paratype); southern shore of Lago Chungará, 18°16’17.0”S; 69°09’37.7”W, 4583 m, FML 26504–508, MCN 4734–35 (paratypes); Provincia de Parinacota, km 12.5 de la ruta andina A93 Parinacota, Visviri, 18°08.115’S, 69°18.000’W, CZZA 346–350 (paratypes); cerca de Putre desvío a Colpita, FML 26509–513 (paratypes).

Liolaemus chiliensis- **ARGENTINA. Provincia de Neuquén.** FML 9451; MVZ 180736-737; 187756-757. MLPR 5241; 5256; MLPS 1742; 1869; 2114. **CHILE. Región de La Araucania.** CMNH 57187; 64719. **Region de Bio Bio.** MCZ 121214-219; 154180-181; 19704-06; 19982-83; 2139; 2537; 65393; MVZ 196545-546; 196759. **Región Metropolitana.** MACN 31363. MACN 11995. **Región Valparaiso.** USNM 64122.

Liolaemus cyanogaster- **ARGENTINA. Provincia de Neuquén.** MVZ 188724; 188725. **CHILE. Región de La Araucania.** SDSU 1833-36. **Región de Los Rios.** AMNH 38065-67; 58343. FML 1671. CMNH 64720-24. **Región de Bio Bio.** MCZ 110468; 126728; MCZ 165179; MCZ 7267-68. FMNH 207036.

Liolaemus exploratorum- **ARGENTINA. Provincia de Santa Cruz.** MLP.S 571; 567; 573; 570.

Liolaemus fuscus- **CHILE**: MACN 16718–23, 21621. **Región de Coquimbo: Coquimbo**, AMNH 131833–834, MCZ 165146. **Región Metropolitana**: El Cerezo, MCZ 65395; La Calera, Aconcagua, MCZ 165150; road to La Disputada, 1.5 km past turn off Forest Los Farellones, 33.33333°S, 70.36667°W, MVZ 187797; on road to La Disputada Mines (33.36667°S, 70.38333°W, MVZ 187804; road to Farellones, 33.35°S, 70.35°W, MVZ 196546–548, 196550, 196559, 196562, 196565, 196574–575, 196581. **Región de Valparaiso**: Bahía Oscuro, FML 1592(2); Parque Nacional Campana, SDSU 1866; Valparaíso, MCZ 38621–626, MCZ 165147.

Liolaemus gracilis- **ARGENTINA. Provincia de Buenos Aires.** MCN 2156-58. MLPR 5306; 1556; 1692. **Provincia de Chubut.** MCN 1345. **Provincia de La Pampa.** FML 8371. **Provincia**

de Mendoza. FML 00963 (3); FML 02731; FML 7234-36; 7238. **Provincia Rio Negro.** FML 2970; FML 8399.

Liolaemus gravenhorsti- **ARGENTINA. Region de Maule.** FML 2255. **Region Metropolitana.** AMNH 80054-055; MCZ 154184-185; MCZ 38627-628; MCZ 65396-397; USNM 165635. MACN 11998-999.

Liolaemus incaicus- **PERU: Departamento de Calca**: near Calca, Hacienda Urco FMNH 266542 (holotype), FMNH 34104, FMNH 34127 (14) (paratypes).

Liolaemus lemniscatus- **CHILE. Region de Bio Bio.** CMNH 64727-728; 64730; CMNH 64729; MCZ 164037-038; 164041; 164045; 164047; 164049; 164056; USNM 58710. **Region de Coquimbo.** FML 1559. **Region Metropolitana.** USNM 165620. FMNH 214220-230.

Liolaemus nitidus- **CHILE. Region de Coquimbo.** MCZ 165447-450. **Region Metropolitana.** FML 1194; FML 1198; MCZ 65402; MCZ 165452; MCZ 165453; MCZ 197708; 19979. **Region de Valparaiso.** CMNH64737. MACN 17315-16.

Liolaemus pagaburoi- **ARGENTINA. Provincia de Tucumán.** FML 16132-33; FML 16838; 1829; FML 2435; FML 2722; 2746; FML 2633;

Liolaemus paulinae- **CHILE: Región de Antofagasta**: Orillas del Rio Loa, Calama, FML 1196 (paratype), 1341 (2); shore of Loa River, SDSU 1909–11, MZUC 19360, 19362–367, 193671, 19370, 19382.

Liolaemus pseudolemniscatus- **CHILE. Region de Coquimbo.** MNHNC 1376-77; 1501; 1531.

Liolaemus puna- **ARGENTINA. Provincia de Jujuy.** FML 929; FML 1265; FML 1512; FML 1519; 1533; 1874; MCN 229-232; MCN 698-99; MCN 1718-19. **Provincia de Salta.** FML 1364; 9914-27; 16611663; 2779; 3647; 3348; 3649; 949-50; SDSU 3579-82. **CHILE. Region de Tarapaca.** SDSU 1697-99; MCZ 149852; 149854-56; 149858; USNM 165641; MZUC 19392. **Region de Atacama.** MNHN 583; 585; 588.

Liolaemus pyriphlogos- **ARGENTINA: Provincia de Jujuy**: vicinity of Laguna Leandro, Humahuaca Department, 23°01’50”S; 65°14’46,8”W, FML 18199 (holotype), FML 18198, 18200–201(paratypes); 10 km antes de Aparzo, desde Humahuaca (23°09’50,5”S, 65°11’48”W), FML 18208–210; afueras de Chorcán, camino a Laguna Leandro, FML 18236; camino a Mudana desde Uquía, (23°20’30”S, 65°13’27,5”W), FML 18250–252; entre Aparzo y Humahuaca (23°10’09.3”S, 65°11’01.4”W), FML 18258–259; entre Chorcán y Laguna Leandro (23°01’57.5”S, 65°14’14.3”W), FML 18260–262; Camino de Humahuaca a Chorcán, 23°10’761”S, 65°11709”W, MCN 226, 228, 589–98; Laguna Leandro, W of Chorcán, Humahuaca, FML 1463 (32); Camino a Laguna Leandro, Humahuaca, FML 3488–89.

Liolaemus ramirezae- **ARGENTINA: Provincia de Catamarca**: Mina Capillitas, Andalgalá, FML 3612; Morro El Arenal, el Ingenio, Andalgalá, FML 2561 (3). **Provincia de Salta**: La Poma, FML 1658, MCN 1733–35; 21 km N de La Poma, FML 3006; Santa Rosa de Tastil, Rosario de Lerma, FML 3335. **Provincia de Tucumán**: km 98, ruta provincial 307, Amaicha del Valle, Tafí del Valle FML 2240, 1367, 2275 (4), 2279 (2), 2330 (4), 2383 (2), 2384–86, 2436, 2463, 2481, 2486; Ruta provincial 307, O de El Infiernillo, Tafi del Valle, FML 2715; km 98.5 ruta provincial 307, Tafi del Valle, FML 8182; km 95 de Ruta provincial 307, Tafi

del Valle, FML 6012, 17438; km 95, Ruta Provincial 307 (26°40.82’S, 65°48.74’W), MCN 466, 469–70.

Liolaemus robertmentensi- **ARGENTINA. Provincia de Catamarca.** Belén, FML 16442, FML 1753 (3), IBIGEO-R 5086-7, MCN 2180. Tinogasta, FML 7710, IBIGEO-R5088, MCN 2610, MLP-S 987.

Liolaemus sanjuanensis- **ARGENTINA: Provincia de San Juan**: Sierra de Pie de Palo, FML 1016 (paratype), UNSJ 735–49, 766.

Liolaemus saxatilis- **ARGENTINA. Provincia de Córdoba.** MCN 903-05; SDSU 1736-37; AMNH 02530-36; 65193-199; 126616; MLP.S 1166-167.

Liolaemus sp1- **BOLIVIA. Departamento La Paz.** CBF 0009; 0085; 2263-2265.

Liolaemus sp2- **BOLIVIA. Departamento Oruro.** CBF 1141-42; 1144; 1145; 1147; 1877-81.

Liolaemus sp3- **BOLIVIA. Departamento La Paz.** CBF 1964; 2278; 2725-29.

Liolaemus sp4-**ARGENTINA. Provincia Neuquen.** BB 578-81; 584; 595.

Liolaemus sp5- **ARGENTINA. Provincia Neuquen.** MCN 907- 908; 911-912.

Liolaemus sp6- **ARGENTINA. Provincia de Santa Cruz.** Los Antiguos, PMC 201, 211, MCN 766, FML 2116 (2), 21305, 21411-415

Liolaemus sp7- **ARGENTINA. Provincia de Santa Cruz.** Lago Posadas, MCN 767-72, PMC 290-93, 733

Liolaemus sp8-**ARGENTINA. Provincia de Neuquen.** FML 07786-787, 07857-858, MCN 216- 217, 906.

Liolaemus sp9- **ARGENTINA. Provincia de Rio Negro.** Ingeniero Jacobacci, FML 21531-33, 10075-076, 16345-346, 21457-458, MACN 15194-196

Liolaemus sp10- **ARGENTINA. Provincia de Rio Negro**. Comallo, FML 10063-067, 21484, 21540-541.

Liolaemus sp11-**ARGENTINA. Provincia de La Rioja.** Anillaco, MACN 24994-997, MCN1755,

Liolaemus sp12-**ARGENTINA. Provincia de Catamarca.** Andalagalá, FML 1308 (5), FML 1478 (3), FML 1482 (4), FML 1616 (7), FML 1706 (5), FML 6403 (2), FML 6601, FML 6786-787, MCN 2611, MCN1713, MCN2571, MCN2574.

Liolaemus sp13- **ARGENTINA. Provincia de La Rioja.** Chilecito, MCN1638-39, MCN1743, MCN1747 – 48; MCN 1754; MCN 1954-57.

Liolaemus sp14- **ARGENTINA. Provincia de Buenos Aires.** Mar del Plata, UNMdP 0565-66 1782-83 1817-18 1823-27

Liolaemus sp15- **Argentina. Provincia de Mendoza.** Malrgüe, FML 7202-13.

Lliolaemus schroederi- **CHILE. Region de Bio Bio.** MCZ 164166; 164169; 164251; MCZ 164299; 164310; 164117; 165079; 165083-84; 165086; 165126; MNHN 2480-81; 2483; 2487. **Region Metropolitana.** USNM 165636.

Liolaemus tacnae- **PERU. Departamento de Arequipa.** FML 1544. **Departamento de Tacna.** MCZ 45806; 45807; 49210-211.

Liolaemus tandiliensis- **ARGENTINA: Provincia de Buenos Aires**: Sierra de los Padres, MCN 1604–05, 1612, 1614–15; Sierra de los Difuntos, MCN 1606–11, 1616–17; Sierra La Brava, MCN 1613.

Liolaemus variegatus- **BOLIVIA. Departamento de Cochabamba.** FML 1210; CBGR 116; 118; 121; 124; 130; 132-39; 145; 150-53; 90-92: 122; MLP.S 841.

Liolaemus walkeri- **PERU. Departamento de Ayacucho.** MCZ 45850; FMNH 81380-387; 81388-389; 81395-396. **Departamento de Junin.** SDSU 1937; AMNH 63389-390; MCZ 43770-775; 43777; MCZ 43779-781; MCZ 45887-888; MCZ 100111; FML 371. **Departamento de Apurimac.** FML 372; 1283. **Departamento de Lima.** MCZ 45783.

Liolaemus yalguaraz- **ARGENTINA: Provincia de Mendoza:** Pampa de Yalguaraz, Departamento Las Heras, 32° 19`59.0” S; 69°22`54.5” W, FML 27622 (holotype); FML 27623-634 (paratypes); MCN 5068-69 (paratypes).

Liolaemus yanalcu- **ARGENTINA. Provincia de Salta.** MCN 1038; 360-61; 541; 702; 705-07; 725-29; 955-60; 1449; 1635; 1750; 2236-39; 2501; 2613-16.

Accession numbers for GeneBank sequences downloaded

Liolaemus alticolor. KF923688.1; KF923660.1

Liolaemus abdalai. JN410525.1; JN410381.1

Liolaemus bibronii. JN410531.1(santa cruz deseado) ; JN410393.1 (santa cruz deseado); JF272767.1(santa cruz deseado) ; JX522215.1(santa cruz deseado) ; JN410371.1 (santa cruz deseado) ; JF272800.1(santa cruz deseado) ; JN410551.1(santa cruz deseado); DQ989784(catan lil neuquen) ; DQ989738.1(pilcaniyeu Rio Negro) DQ989652 (malargue mendoza); DQ989768 (San Rafael, Mendoza); DQ989754.1 (cuschamen, Chubut);

Liolaemus bitaeniatus. AY662062

Liolaemus chaltin. AY662061

Liolaemus chavin. KF923683.1; KF923655.1; KF923682.1; KF923654.1; KF923682.1; KF923654.1; KF923681.1; KF923653.1

Liolaemus chiliensis. DQ989785; EU649245.1; EU649304.1

Liolaemus cyanogaster. EU649247.1; EU649306.1; EU649246.1; EU649305.1

Liolaemus gracilis. DQ989662; JN410544.1; JN410380.1; JN410554.1; JN410399.1;

JN410538.1; JN410397.1

Liolaemus incaicus. KF923685.1; KF923657.1

Liolaemus lemniscatus. EU649104.1; EU649140.1; EU649119.1; EU649155.1; EU649132.1; EU649125.1; EU649160.1

Liolaemus pachacutec. KF923663.1; KF923635.1; KF923662.1; KF923634.1;

KF923661.1; KF923633.1

Liolaemus pagaburoi. AY662058

Liolaemus pseudolemniscatus. EU220833

Liolaemus puna. AY662059

Liolaemus ramirezae. DQ237715.1; DQ237597.1; JN410520.1; JN410394.1; DQ989772

Liolaemus robertmertensi. JN410535.1; JN410398.1

Liolaemus saxatilis. JN410553.1; JN410365.1; JN410553.1; JN410365.1

Liolaemus sp 12. DQ989769.1; DQ237499.1

Liolaemus tacnae. KF923664.1; KF923636.1; KF923671.1; KF923643.1; KF923670.1; KF923642.1; KF923669.1; KF923641.1; KF923668.1; KF923640.1; KF923667.1; KF923639.1; KF923666.1; KF923638.1; KF923665.1; KF923637.1

Liolaemus walkeri. KF923677.1; KF923649.1; KF923674.1; KF923646.1; KF923672.1; KF923644.1; KF923675.1; KF923647.1; KF923673.1; KF923645.1; AY662057.1

Liolaemus wari. KF923680.1; KF923652.1; KF923679.1; KF923651.1; KF923678.1; KF923650.1

Liolaemus yanalcu. AY662056

Liolaemus multicolor. KF969085.1; KF968893.1

Liolaemus parvus. AY173610.1; AY367839.1
