# Supplementary material for: Phylogeny, time divergence, and historical biogeography of the South American Liolaemus alticolor-bibronii group (Iguania: Liolaemidae)
Source: PeerJ. 2018 Feb 20;6:e4404. doi: 10.7717/peerj.4404 (PMC5824678; doi:10.7717/peerj.4404)
Supplement: File S2 [file peerj-06-4404-s002.pdf]

Supplemental File S2

List of morphological character employed in the Phylogenetic Analyses.

Variation and character states for discrete characters are showed between parentheses. NPB=non polymorphic binary; PB= polymorphic binary; NPM= non polymorphic multistate; PM= polymorphic multistate.

CONTINUOUS CHARACTERS

1. Number of scales around midbody
2. Number of scales between occiput and thighs
3. Number of scales in dorsal head, from occiput to rostral (Hellmich Index)
4. Number of supercilliaries
5. Number of scale organs in postrostrals
6. Number of temporal scales
7. Number of neck's scales
8. Number of scales of neck, from posterior margin of auditory meatus to antehumeral fold.
9. Number of gular scales
10. Number of upper cilliaries scales
11. Number of lower cilliaries scales
12. Number of lorilabials scales
13. Number of lorilabials scales contactoing subocular
14. Number of supralabials
15. Number of infralabials
16. Number of postmentals (Chinshields)
17. Number of ventrals
18. Number of precloacal pores in males
19. Number of precloacal pores in females
20. Number of digital lamellae on finger 1
21. Number of digital lamellae on finger 2
22. Number of digital lamellae on finger 3
23. Number of digital lamellae on finger 4
24. Number of digital lamellae on finger 5
25. Length of finger 1/SVL
26. Length of finger 2/SVL
27. Length of finger 3/SVL
28. Length of finger 4/SVL
29. Length of finger 5/SVL
30. Number of digital lamellae on toe 1
31. Number of digital lamellae on toe 2
32. Number of digital lamellae on toe 3

33. Number of digital lamellae on toe 4
34. Number of digital lamellae on toe 5
35. Length of toe 1/SVL
36. Length of toe 2/SVL
37. Length of toe 3/SVL
38. Length of toe 4/SVL
39. Length of toe 5/SVL
40. Head Length/SVL in males
41. Head Length/SVL in females
42. Head width/SVL in males
43. Head width/SVL in females
44. Head heigth/SVL in males
45. Head heigth/SVL in females
46. Trunk length/SVL in males
47. Trunk length/SVL in males
48. Humerus length/SVL
49. Tibia length/SVL
50. Foot length/SVL in males
51. Foot length/SVL in females
52. Tail length/SVL
53. Wide of tail base /SVL in males
54. Wide of tail base /SVL in males
55. Distance between posterior edge of the eye and the anterior edge of the auditory meatus /head length
56. Height of auditory meatus /wide of auditory meatus
57. Length of fourth supralabial/head length
58. Internares distance/head width
59. Interorbital distance/head width
60. Length of subocular/head length
61. Length of fourth lorilabial/head length
62. Length of preocular/head length
63. Length of posocular/head length
64. Length of auricular/head length

DISCRETE CHARACTERS

65. Shape of dorsal scales of body (PM; 0=rounded/1=lanceolate/2=rombhoidal)
66. Disposition of dorsal scales of body (NPM; 0=juxtaposed/1=slightly imbricated/2= strongly imbricated)

67. Surface of dorsal scales of body – Presence of keel (NPM; 0=smooth, without keel/1= slightly keeled/2=strongly keeled)
68. Point shaped edge of dorsal scales of body (mucronated) (NPB; 0=not mucronated/1=slightly mucronated/2= strongly mucronated)
69. Surface of scales of dorsal head (PB; 0=smooth/1= slightly rugouse/2= rugouse)
70. Supraorbital semicircles (PB; 0=complete/1=incomplete)
71. Number of supraocular scales (PB; 0= more than four/1= four or less)
72. Number of enlarged supraocular scales (PB; 0= more than three/1= three or less)
73. Fragmentation of frontal scale (PB; 0 = fragmented/1= azygous, not fragmented).
74. Interparietal size vs parietal size (PM; 0=smaller/1= equal/2= bigger)
75. Contact between nasal and rostral (PM; 0= without contact, separated by one scale/ 1= narrow contact/2= broad contact)
76. Number of scales between canthal and nasal (NPB; 0= one scale/1= two scales)
77. Number of scales between subocular and nasal (PB; 0= four or more scales/1= three scales)
78. Number of scales between subocular and canthal (PM; 0= five/1=four/2= three/3= two)
79. Preocular contacting lorilabial row (PB; 0= Without contact; 1= contacting)
80. Shape of supralabials (NPB; 0= wide, bigger than lorilabials; 1= narrow, equal in size than lorilabials)
81. Fourth supralabial upturned (NPB; 0= not upturned/1= upturned)
82. Fourth supralabial contacting subocular (PB; 0= there are not contact/1= contacting),
83. Surface of temporal scales (PMS; 0= smooth/1= slightly keeled/2= strongly keeled)
84. Auricular scale (PB; 0=Absent/1=Present).
85. Inferior edge of the auricular scale (NPB; 0= don't reach the half line of the auditory meatus/1= reach or exceed the half line of auditory meatus)
86. Enlarged scales on anterior edge of auditory meatus (NPB; 0= Present/1= absent)
87. Shape of neck scales (PB; 0= granular/1= laminar)
88. Surface of neck scales (BP; 0= smooth/1= slightly keeled/2= strongly keeled)
89. Number of scales contacting mental sclae (PB; 0= four/1= more than four)
90. Number of posmental scales (Chinshields) (PM; 0= three/1= four/2=five)
91. Number of scales between posmental II (PB; 0= two or more scales/1= one scale)
92. Number of scales between posmental III (PB; 0= four or more scales/1= three or less scales)
93. Number of scales between posmental IV (PB; 0= more than five scales/1= five or less scales)
94. Pigmentation on dorsal head (PM; 0= irregular spots/1= melanic/2= without spots)
95. Black line surrounding interparietal (PB; 0=absent/1=Present)
96. Black line extending forward from interparietal scale to frontonasal scales (PB; 0= Absent/1= Present)
97. Subocular whitish (PB; 0= no, same color as loreal region/1= yes, lighter than loreal region)
98. Band-shaped spot in middle region of subocular (PB; 0= absent/ = present).
99. Band-shaped spot in posterior region of subocular (P; 0= absent/1= present)

100. Line or light stripe which begins in postero-superior edge of the eye, and reaches dorsolateral stripes (PB; 0= absent/1= present)
101. Black line bordering the line or light stripe that begins in postero-superior edge of the eye, and reaches dorsolateral stripes (PB; 0= absent/1= present)
102. Line or light stripe that begins at the middle of posterior edge of the eye, and extends back to upper edge of auditory meatus (PB; 0= absent/1= present)
103. Black line bordering the line or light stripe that begins at the middle of posterior edge of the eye, and extends back to upper edge of auditory meatus (PB; 0= absent/1= present)
104. Line or light stripe that begins at posterior of edge auditory meatus and extends to shoulder, along the auricular and longitudinal folds (PB; 0= absent/1= present)
105. Black line bordering the line or light stripe that begins at posterior edge of auditory meatus and extends to shoulder, along the auricular and longitudinal folds (PB; 0= absent/1= present)
106. White line in rictal fold (PB; 0= absent/1= present)
107. Paravertebral spots in males (PB; 0= present/1= absent)
108. Paravertebral spots in females (PB; 0= present/1= absent)
109. Shape of paravertebral spots (PM; 0= Absent/1=rectangular/2= rounded/3= lines perpendicular to body axis/4= lines parallel to body axis/5= dot shaped/6= U shaped/7= triangular)
110. White edge of paravertebral spots (PB; 0= absent/1= present)
111. Dorsolateral stripes in males (PB; 0= Absent/1= present)
112. Dorsolateral stripes in females (PB; 0= Absent/1= present)
113. Black margin of dorsolteral stripes (PB; 0= Absent/1= Present)
114. Number of scales included in the wide of dorsolateral stripes (NPMS, 0=0/1= 0.5-1/2=1.5-2/3=2.5-3)
115. Vertebral line in females (PB; 0= Absent/1= Present)
116. Vertebral line in males (PB; 0= Absent/1= Present)
117. Background color of lateral field (PB; 0= same color than dorsum/1= darker tan dorsum).
118. Spots in lateral field in females (PM; 0= spotless/1= black spots/2= white spots)
119. Spots in lateral field in males (PM; 0= spotless/1= black spots/2= white spots)
120. Shape of spots in lateral fields in females (PM; 0= spotless/1= rounded/2= irregular)
121. Shape of spots in lateral fields in males (PMS 0= spotless/1= rounded/2= irregular)
122. Lines in lateral field in females (PM; 0= without lines/1= black lines/2= white lines/3= light blue lines/4= orange or yellow lines)
123. Lines in lateral field in males (PM; 0= without lines/1= black lines/2= white lines/3= light blue lines/4= orange or yellow lines)
124. Shape of lines in lateral field in females (NPM; 0= without lines/1= narrow/2= broad – band-shaped)
125. Shape of lines in lateral field in males (NPM; 0= without lines/1= narrow/2= broad – band-shaped)
126. Iridescent scales on flaks of body (PB; 0= present/1= absent)
127. Ventrolateral line (PB; 0= absent/1= Present)

128. Background color in ventrolateral region (PM; 0= Light blue or orange/1= cream color/2= gray /3= melanic)
129. Color pattern in ventrolateral region (PM; 0=without pattern/1= lines/2= black spots/3= white spots)
130. Background color of throat in females (PM; 0= cream color/1= light gray/2= lead gray/3= melanic/4= blue)
131. Background color of throat in males (PM; 0= cream color/1= light gray/2= lead gray/3= melanic/4= blue)
132. Color pattern of throat in females (PM; 0= variegated/1= irregular spots/2= without pattern)
133. Color pattern of throat in females (PM; 0= variegated/1= irregular spots/2= without pattern/ 3= white spots)
134. Background color of chest in females (PM; 0= cream color/1= gray/2= melanic/3= blue)
135. Background color of chest in females (PM; 0= cream color/1= gray/2= melanic/3= blue)
136. Color pattern in chest (PM; 0= variegated/1= spots/2= without pattern)
137. Background color of belly (PM; 0= cream color/1= gray/2= melanic/3= blue)
138. Color pattern of belly (PM; 0= variegated/1= spots/2= without pattern)
139. Vertebral line in tail (PB; 0=Absent; 1=Present)
140. Dorsolateral stripes in tail (NPB; 0= Absent/1= Present)
141. Paravertebral spots in tail (NPB; 0= Absent/1= Present)
142. Shape of paravertebral spots of tail (NPM; 0 = rounded/1= lines perpendicular to body axis/2= rectangular)
143. Ringed pattern in tail (NPB: 0= present/1= absent)
144. Ventral background color of tail in females (NPB; 0= cream color/1= gray/2=melanic)
145. Ventral background color of tail in females (NPB; 0= cream color/1= gray/2=melanic)
146. Ventral color pattern (NPM; 0= irregular spots/1= lines/2= without pattern)
147. Number of rows in which the scale organs of third lorilabial are arranged (PM; 0=three rows/1= two rows/2= one row)
148. Number of rows in which the scale organs of the scale located under the preocular and canthal are arranged (PM; 0= one row/1= two rows/2= three rows)
149. Antegular fold (PB; 0= present/1=absent)
150. Neck folds Y-shaped (PB; 0= present /1= absent)
151. Horizontal fold (PB; 0= present /1= absent)
152. Precloacal pores in females (PB; 0= absent/1= present)
153. Microhabitat preferences (PM; 0= saxicolous/1= terrestrial, *Festuca* sp./2= terrestrial, bushes/3= terrestrial, logs)
154. Reproductive mode (NPB; 0= viviparous/1= oviparous)
155. Reproductive coloration in females (NPB; 0= absent/1= present)
156. A blade-like tibial process (NPB; 0= present/1= absent)
157. Meckel’s groove (NPB; 0= open/1= fused)
158. Number of scales between the frontal and supercilliaries (PB; 0= four or less/1= five or more)

159. Number of scales between frontal and rostral (PB; 0= five or less/1= six or more)
160. Number of scales contacting interparietal (PB; 0= six or less/1= seven or more)
161. Number of scales forming the supraorbital semicircles (NPM; 0=6-8/1=9-10/2= 1-12)
162. Number of scales surroundig nasal (NPB; 0= six/1= seven or more)
163. Snout-Vent-Length in females (NPM; 0=36-45/1=45.1-58/2=58.1-65)
164. Snout-Vent-Length in males (NPM; 0=44-53/1=53.1-62/2=62.1-70)
165. Wide of vertebral region in mm (NPM; 0=0, without vertebral region/1= 0-1.72/2= 1.73-3.3/3= 3.6-4.68/4= 5 or more)
166. Percentage of females exhibiting precloacal pores (NPM; 0=0%/1=1-36.5%/2=37-65%/3=66-100%)
167. Background color of lateral field (NPM; 0= light brown/1= dark brown/2= orange/3= gray; 4= melanic/5= yellow/6= green/7= red)
