# Supplementary material for: Phylogeny, time divergence, and historical biogeography of the South American Liolaemus alticolor-bibronii group (Iguania: Liolaemidae)
Source: PeerJ. 2018 Feb 20;6:e4404. doi: 10.7717/peerj.4404 (PMC5824678; doi:10.7717/peerj.4404)
Supplement: File S3 [file peerj-06-4404-s003.pdf]

Supplemental File S3

Trees of DEC analysis found with the constrained adjacency matrix.

S3.1. Tree found with dispersal cost Matrix I

S3.2 Tree found with dispersal cost Matrix II

S3.3 Tree found with dispersal cost Matrix III

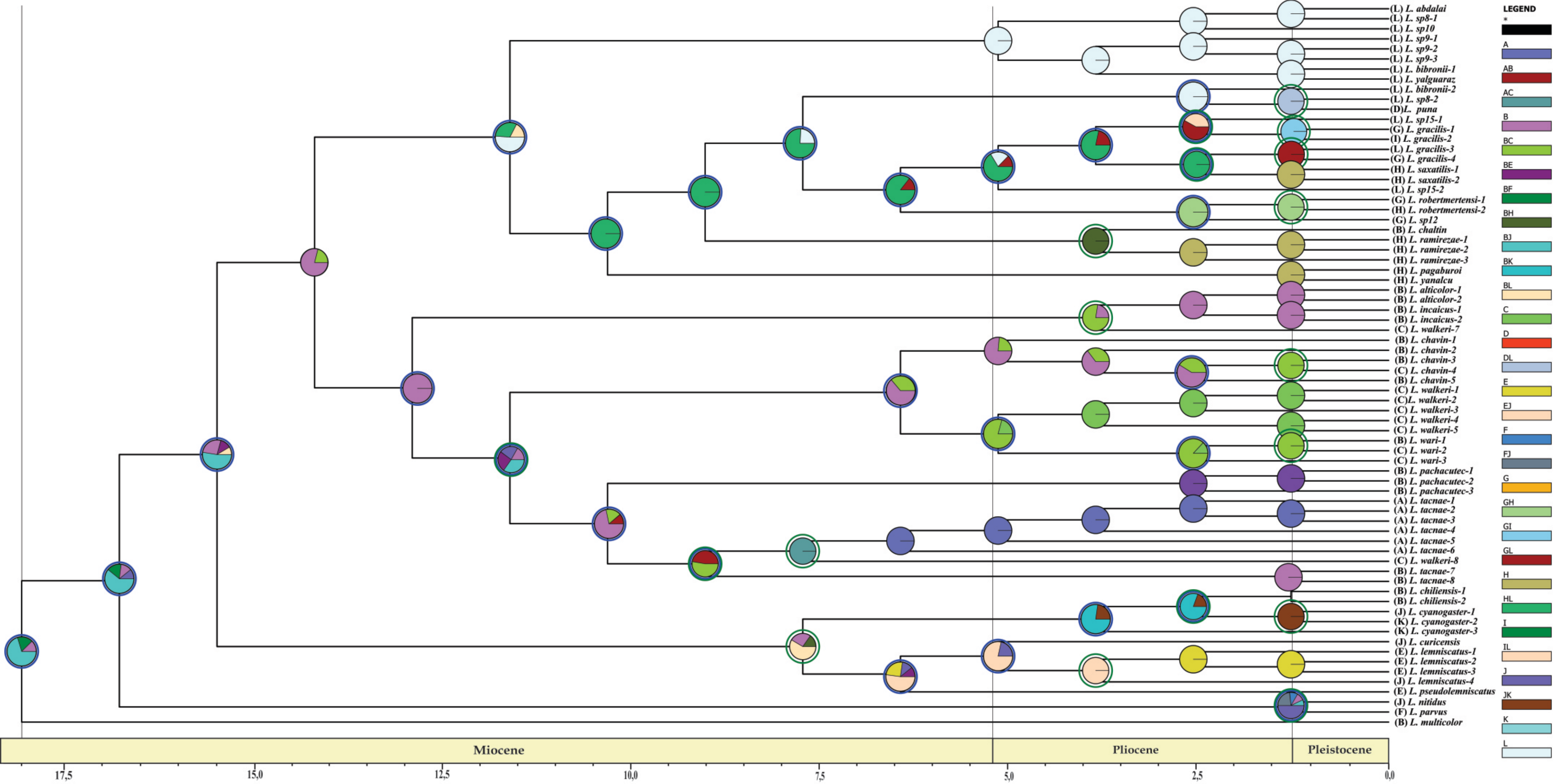
(L) L. abdalai
(L) L. sp8-1
(L) L. sp10
(L) L. sp9-1
(L) L. sp9-2
(L) L. sp9-3
(L) L. bibronii-1
(L) L. yalguaraz
(L) L. bibronii-2
(L) L. sp8-2
(D)L. puna
(L) L. sp15-1
(G) L. gracilis-1
(I) L. gracilis-2
(L) L. gracilis-3
(G) L. gracilis-4
(H) L. saxatilis-1
(H) L. saxatilis-2
(L) L. sp15-2
(G) L. robertmertensi-1
(H) L. robertmertensi-2
(G) L. sp12
(B) L. chaltin
(H) L. ramirezae-1
(H) L. ramirezae-2
(H) L. ramirezae-3
(H) L. pagaburoi
(H) L. yanalcu
(B) L. alticolor-1
(B) L. alticolor-2
(B) L. incaicus-1
(B) L. incaicus-2
(C) L. walkeri-7
(B) L. chavin-1
(B) L. chavin-2
(B) L. chavin-3
(C) L. chavin-4
(B) L. chavin-5
(C) L. walkeri-1
(C)L. walkeri-2
(C) L. walkeri-3
(C) L. walkeri-4
(C) L. walkeri-5
(B) L. wari-1
(C) L. wari-2
(C) L. wari-3
(B) L. pachacutec-1
(B) L. pachacutec-2
(B) L. pachacutec-3
(A) L. tacnae-1
(A) L. tacnae-2
(A) L. tacnae-3
(A) L. tacnae-4
(A) L. tacnae-5
(A) L. tacnae-6
(C) L. walkeri-8
(B) L. tacnae-7
(B) L. tacnae-8
(B) L. chiliensis-1
(B) L. chiliensis-2
(J) L. cyanogaster-1
(K) L. cyanogaster-2
(K) L. cyanogaster-3
(J) L. curicensis
(E) L. lemniscatus-1
(E) L. lemniscatus-2
(E) L. lemniscatus-3
(J) L. lemniscatus-4
(E) L. pseudolemniscatus
(J) L. nitidus
(F) L. parvus
(B) L. multicolor
LEGEND
*
A
AB
AC
B
BC
BE
BF
BH
BJ
BK
BL
C
D
DL
E
EJ
F
FJ
G
GH
GI
GL
H
HL
I
IL
J
JK
K
L
Miocene
Pliocene
Pleistocene
17,5
15,0
12,5
10,0
7,5
5,0
2,5
0,0

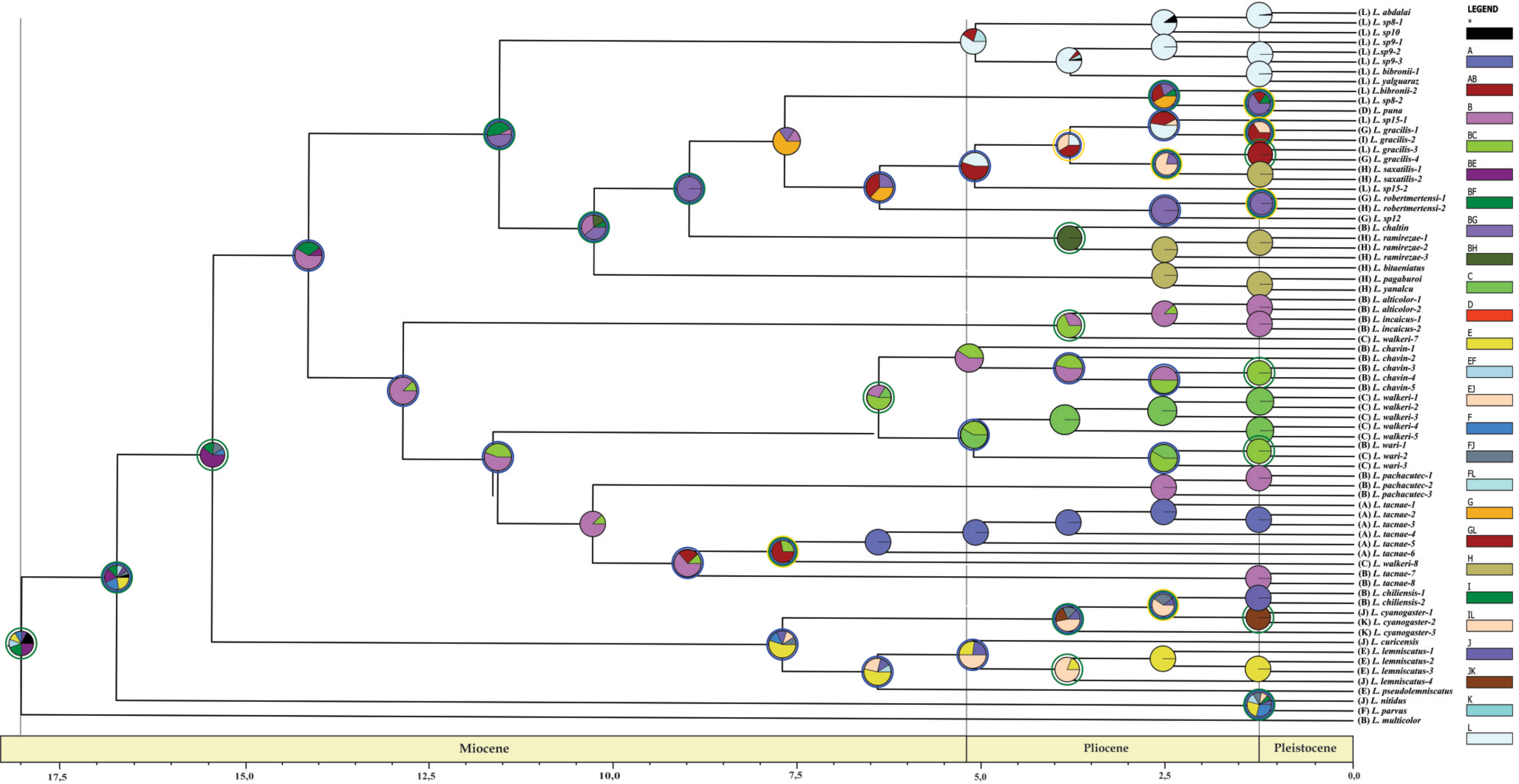

(L) L. abdalai
(L) L. sp8-1
(L) L. sp10
(L) L. sp9-1
(L) L.sp9-2
(L) L. sp9-3
(L) L. bibronii-1
(L) L. yalguaraz
(L) L.bibronii-2
(L) L. sp8-2
(D) L. puna
(L) L. sp15-1
(G) L. gracilis-1
(I) L. gracilis-2
(L) L. gracilis-3
(G) L. gracilis-4
(H) L. saxatilis-1
(H) L. saxatilis-2
(L) L. sp15-2
(G) L. robertmertensi-1
(H) L. robertmertensi-2
(G) L. sp12
(B) L. chaltin
(H) L. ramirezae-1
(H) L. ramirezae-2
(H) L. ramirezae-3
(H) L. bitaeniatus
(H) L. pagaburoi
(H) L. yanalcu
(B) L. alticolor-1
(B) L. alticolor-2
(B) L. incaicus-1
(B) L. incaicus-2
(C) L. walkeri-7
(B) L. chavin-1
(B) L. chavin-2
(B) L. chavin-3
(B) L. chavin-4
(B) L. chavin-5
(C) L. walkeri-1
(C) L. walkeri-2
(C) L. walkeri-3
(C) L. walkeri-4
(C) L. walkeri-5
(B) L. wari-1
(C) L. wari-2
(C) L. wari-3
(B) L. pachacutec-1
(B) L. pachacutec-2
(B) L. pachacutec-3
(A) L. tacnae-1
(A) L. tacnae-2
(A) L. tacnae-3
(A) L. tacnae-4
(A) L. tacnae-5
(A) L. tacnae-6
(C) L. walkeri-8
(B) L. tacnae-7
(B) L. tacnae-8
(B) L. chiliensis-1
(B) L. chiliensis-2
(J) L. cyanogaster-1
(K) L. cyanogaster-2
(K) L. cyanogaster-3
(J) L. curicensis
(E) L. lemniscatus-1
(E) L. lemniscatus-2
(E) L. lemniscatus-3
(J) L. lemniscatus-4
(E) L. pseudolemniscatus
(J) L. nitidus
(F) L. parvus
(B) L. multicolor
LEGEND
*
A
AB
B
BC
BE
BF
BG
BH
C
D
E
EF
EJ
F
FJ
FL
G
GL
H
I
IL
J
JK
K
L
Miocene
Pliocene
Pleistocene
17,5
15,0
12,5
10,0
7,5
5,0
2,5
0,0

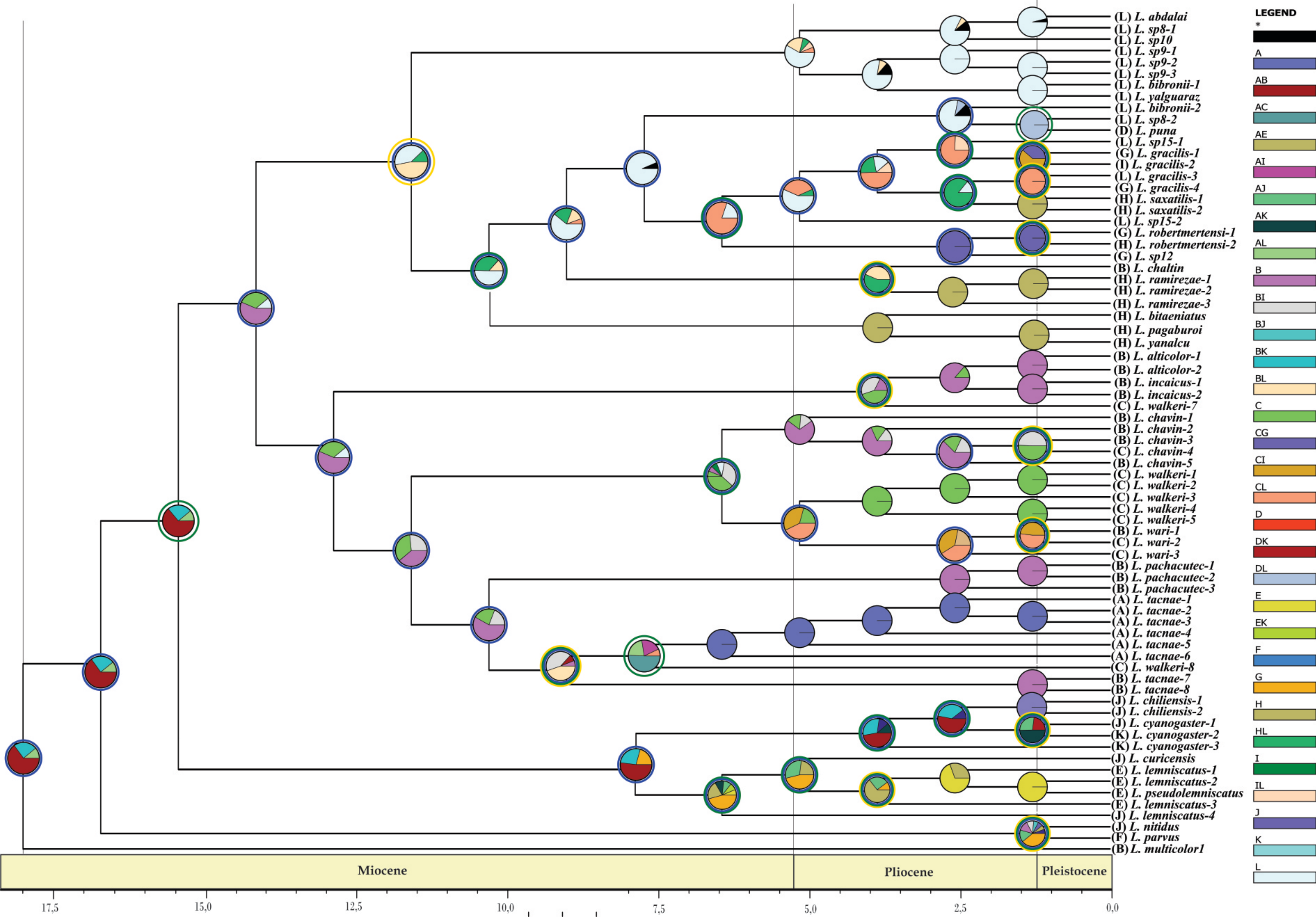
(L) L. abdalai
(L) L. sp8-1
(L) L. sp10
(L) L. sp9-1
(L) L. sp9-2
(L) L. sp9-3
(L) L. bibronii-1
(L) L. yalguaraz
(L) L. bibronii-2
(L) L. sp8-2
(D) L. puna
(L) L. sp15-1
(G) L. gracilis-1
(I) L. gracilis-2
(L) L. gracilis-3
(G) L. gracilis-4
(H) L. saxatilis-1
(H) L. saxatilis-2
(L) L. sp15-2
(G) L. robertmertensi-1
(H) L. robertmertensi-2
(G) L. sp12
(B) L. chaltin
(H) L. ramirezae-1
(H) L. ramirezae-2
(H) L. ramirezae-3
(H) L. bitaeniatus
(H) L. pagaburoi
(H) L. yanalcu
(B) L. alticolor-1
(B) L. alticolor-2
(B) L. incaicus-1
(B) L. incaicus-2
(C) L. walkeri-7
(B) L. chavin-1
(B) L. chavin-2
(B) L. chavin-3
(C) L. chavin-4
(B) L. chavin-5
(C) L. walkeri-1
(C) L. walkeri-2
(C) L. walkeri-3
(C) L. walkeri-4
(C) L. walkeri-5
(B) L. wari-1
(C) L. wari-2
(C) L. wari-3
(B) L. pachacutec-1
(B) L. pachacutec-2
(B) L. pachacutec-3
(A) L. tacnae-1
(A) L. tacnae-2
(A) L. tacnae-3
(A) L. tacnae-4
(A) L. tacnae-5
(A) L. tacnae-6
(C) L. walkeri-8
(B) L. tacnae-7
(B) L. tacnae-8
(J) L. chiliensis-1
(J) L. chiliensis-2
(J) L. cyanogaster-1
(K) L. cyanogaster-2
(K) L. cyanogaster-3
(J) L. curicensis
(E) L. lemniscatus-1
(E) L. lemniscatus-2
(E) L. pseudolemniscatus
(E) L. lemniscatus-3
(J) L. lemniscatus-4
(J) L. nitidus
(F) L. parvus
(B) L. multicolor1
LEGEND
*
A
AB
AC
AE
AI
AJ
AK
AL
B
BI
BJ
BK
BL
C
CG
CI
CL
D
DK
DL
E
EK
F
G
H
HL
I
IL
J
K
L
Miocene
Pliocene
Pleistocene
17,5
15,0
12,5
10,0
7,5
5,0
2,5
0,0
