# Supplementary figures and images for: Phylogeny, time divergence, and historical biogeography of the South American Liolaemus alticolor-bibronii group (Iguania: Liolaemidae)
Source: PeerJ. 2018 Feb 20;6:e4404. doi: 10.7717/peerj.4404 (PMC5824678; doi:10.7717/peerj.4404)

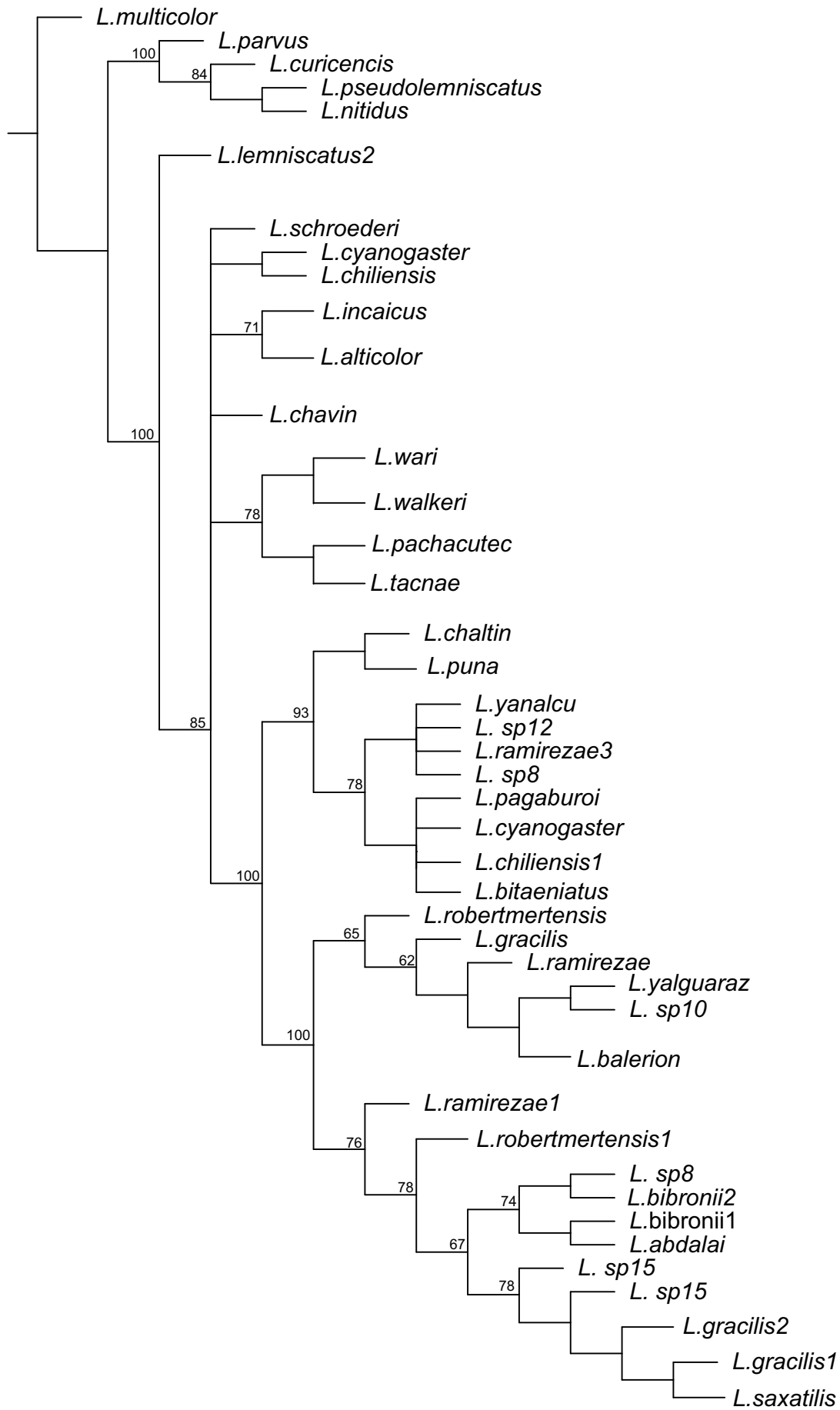

Supplement: File S4 [file peerj-06-4404-s004.pdf]

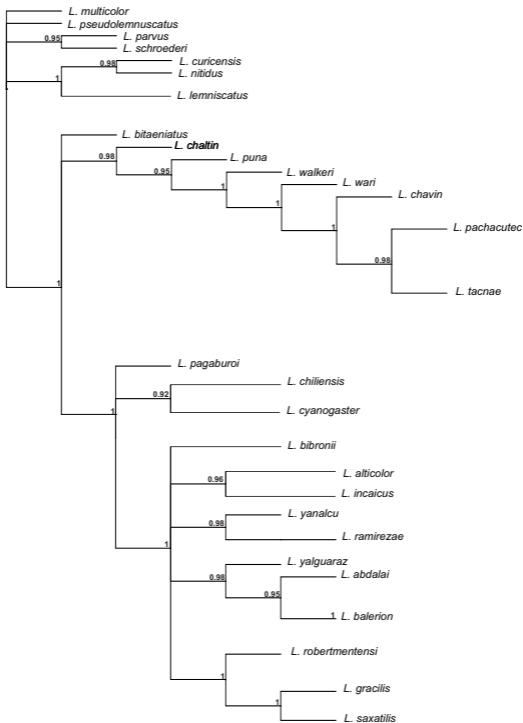

Supplement: File S5 [file peerj-06-4404-s005.pdf]

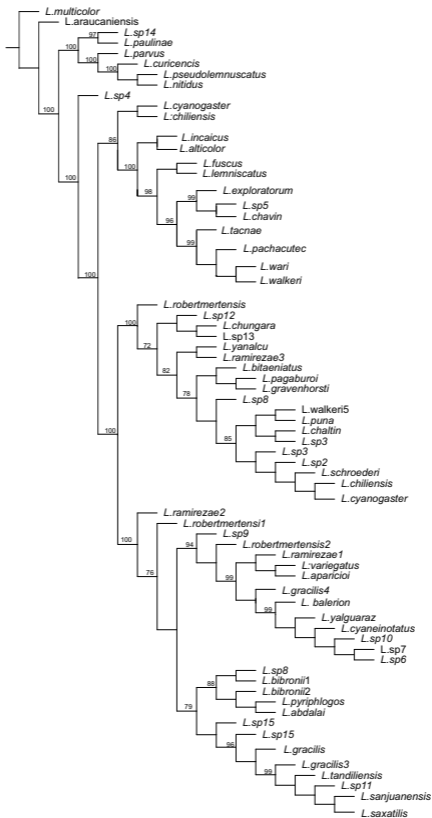

Supplement: File S6 [file peerj-06-4404-s006.pdf]
